# Twist measurement of the left ventricle through radial tagging

**DOI:** 10.1186/1532-429X-15-S1-E48

**Published:** 2013-01-30

**Authors:** Razieh Kaveh, Abbas N Moghaddam, Sarah N Khan, J Paul Finn

**Affiliations:** 1 Biomedical Engineering, Tehran Polytechnic, Tehran, Islamic Republic of Iran; 2 Radiological Science, David Geffen School of Medicine at UCLA, Los Angeles, CA, USA

## Background

Assessment of left ventricular twist is of fundamental importance in evaluating cardiac pump function. The recently developed radial tagging method provides new insights into cardiac rotational motion and has facilitated quantifying LV twist. Herein, we suggest a method to simply obtain twist of radially tagged LV, directly in the image domain.

## Methods

Short axis radially tagged images were acquired at apical and basal levels in 6 healthy subjects.

Contouring LV epicardial and endocardial borders is the only user interaction in the twist extraction process. Applying a proper threshold algorithm over the masked images results in binary images containing easily detectable tags. We tracked the mean position of tags over the cardiac cycle and utilized their spatial coordinates to calculate rotation by the deformation gradient tensor method. By calculating the rotation angle of apical and basal LV sections, we extract twist, as the difference of apical and basal rotation.

## Results

**Figure 1:**
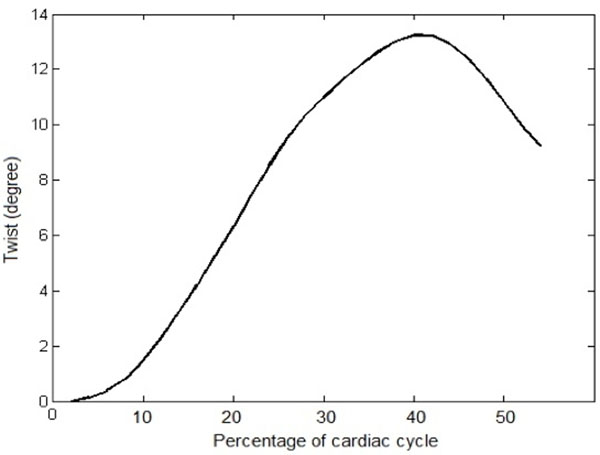
Mean LV twist obtained by proposed method in 6 healthy subjects

The mean twist extracted from 6 healthy subjects is plotted in Figure 1. The values of peak twist and peak twist time are shown in Table 1. Compared to global circumferential strain, obtained with the method reported in [1], peak twist and peak systolic strain occur at the same time for all 6 datasets. The results are consistent with those reported in the literature.

**Table 1:** The values of peak twist and peak twist time

	Volunteer1	Volunteer2	Volunteer3	Volunteer4	Volunteer5	Volunteer6
Peak twist time (percantage of cardiac cycle)	%44	%43.8	%46.7	%40	%45	%50

Peak twist value (degree)	8.8	11.9	13.2	17.3	14.9	16.2

## Conclusions

The proposed method takes advantages of novel radial tagging and is fast and reliable to quantify LV twist, providing additional insight into cardiac contractile function. As derived quantities, other related indices such as torsion, twisting rate and untwisting rate can be calculated.

## Funding

Images were obtained through DCVI section at UCLA.

